# Older Workers and Care-Giving in England: the Policy Context for Older Workers’ Employment Patterns

**DOI:** 10.1007/s10823-017-9332-6

**Published:** 2017-08-04

**Authors:** Sue Yeandle, Lisa Buckner

**Affiliations:** 1 Department of Sociological Studies, University of Sheffield, Elmfield, Northumberland Road, Sheffield, S10 2TU UK; 2 School of Sociology and Social Policy, University of Leeds, Leeds, UK

**Keywords:** Carers, Employment, Older workers, Work-care reconciliation

## Abstract

This article considers recent changes in the incidence of caring among people aged 50-64 in England and the policy context in which these have occurred. After introducing the topic, research questions addressed and methods used, it outlines findings from other research on how older workers experience and manage caring roles. It then sets out relevant public policy developments since carers were first accorded rights to recognition and services in 1995, focusing on workplace support, local services and financial help for people who reduce or quit their paid work to care. The article presents new analyses of the population censuses conducted in England in 2001 and 2011, focusing on people aged 50-64 and especially on those aged 60-64, the group in which the largest changes were seen. Theses show growth in caring at higher levels of intensity for older workers, and increases in the incidence of caring alongside paid work. To deepen understanding of these changes, the analysis also draws on data from a government survey of carers conducted in 2009-10. The concluding discussion argues that although the modest policy changes implemented since 1995 have provided some support to older workers managing work and care, more policy attention needs to be given following the sharp increase in the incidence of caring seen among people aged 50-64 in England between 2001 and 2011.

## Introduction

In this article we consider the incidence of unpaid caring for older, sick and disabled people among men and women aged 50–64 in England between 2001 and 2011, setting this in its policy context and drawing particular attention to carers aged 60–64, who in 2001 had been born in 1937–41, and in 2011 were ‘baby-boomers’ born in 1947–51.

The article focuses on the relationship between caring responsibilities and employment and considers some of the factors which shape people’s labour force participation in years when many are at the peak of their career or earning capacity. The 50–64 age group is of particular interest as this is the age when caring responsibilities for older or disabled family members or friends are most likely to occur, and because England saw rising employment rates among men and women of this age in the period studied. The article makes two contributions. First, it presents a new analysis of policy developments affecting carers^1^ since 1995, when legislation on their recognition and entitlements was first enacted in the UK, highlighting evidence of its consequences using available official data and considering three types of policy development relevant to work, care and older workers: workplace support; local services; and financial help for people who reduce their paid work to care, or cannot enter paid work because of their caring role. Second, it presents new statistical analysis of care responsibility and labour force participation in later working life, extending previous understanding by examining census and government survey data on carers in specific age groups.

The data on carers and employment analysed here are for a decade (2001–11) which began after the *Carers [Recognition and Services] Act 1995* gave carers a legal status, introduced the concept of a carer’s assessment, and gave some the right to a local authority assessment of their needs. Shortly after, the incoming Labour Government introduced a cross-departmental ‘National Carers’ Strategy’ (NCS) and acknowledged carers’ difficulties in reconciling work and care and their need for services and support (HMG 1999).^2^ Midway through our period, a revised NCS (again under a Labour administration) focused more specifically on the needs of carers who wished to combine work and care, also addressing their health, incomes and rights (HMG 2008). This made some new public funding commitments, and indicated how policy in support of carers should develop by 2018 (HMG, 2008). Following the general election of 2010, the incoming Conservative-Liberal Democrat Coalition Government signalled a similar approach, identifying ‘*enabling carers to fulfil their educational and employment potential*’ as a priority in this area (HMG 2010: 5). (As described in the discussion, the *Care Act 2014* later consolidated much earlier legislation into a single statute and gave some carers the right to have their assessed needs met through publicly funded services.)

Levels of employment in the general population of older workers aged 50–64 changed during this period (Smeaton et al. 2009). Employment rates for women of this age had been increasing for some years when the *Carers (Recognition and Services) Act 1995* was passed, and continued on an upward trend thereafter. The employment rates of older men, which had been falling for several decades, also began to rise in the late 1990s (Yeandle 2005). At this time, new policy and financial pressures on workers in their fifties and sixties were encouraging them to remain active in the labour force: an increase in state pension age was legislated for in the *Pensions Acts* of 1995 and 2007 (Bozio et al. 2010; Cribb et al. 2014) and after the global financial crisis of 2008–09, a period of economic austerity, pay freezes and welfare cut-backs also encouraged older workers to retire later (Altman 2014).

The wider context for the analysis in this article is population ageing, the increased longevity of people of all ages with serious illness or disability, and growing evidence that it is unpaid family or friend carers who provide most of the care needed by older, sick and disabled people (National Audit Office 2014). These are issues we have considered elsewhere and which have been widely discussed in the literature (Yeandle & Buckner 2007; CSCI 2009; Buckner et al. 2013). Here we explore how older workers’ participation in paid work changed over a decade for which reliable and detailed statistical data is available, in a period in which the question of how paid work and caring roles can be reconciled received growing attention in public and employment policy. In our concluding discussion we comment on the adequacy and ongoing impact of the policy developments outlined as supports for older workers combining work and care.

## Method and Data

Our method comprises two elements: an examination of policy developments and their consequences, and new statistical analysis of official data.

The policy analysis explores how public and employment policy affecting carers developed in the period under study, and is based on examination of published government policy documents, national legislation, and public statements made by organisations concerned with carers and employment; it is also informed by one author’s membership of relevant expert and advisory groups and committees in the period.^3^

The statistical analysis, using official data, addresses three specific questions: How did patterns of caring and labour force participation change for people aged 50–64 between 2001 and 2011? How were these patterns affected by gender and family relationships? How did changes in caring and employment in this decade vary by intensity of caring? To address the second of these, our new analysis of census data on carers is supplemented with new analysis of the official *Survey of Carers in Households 2009–10* (SCH).

To explore the three questions, we use the national Censuses of Population collected in England in April 2001 and April 2011 to highlight changes in the relationship between work and care for people aged 50–64 between 2001 and 2011. All residents were legally obliged to respond to these, answering questions which in both years included: ‘*Do you look after, or give any help or support to family members, friends, neighbours or others because of either: long-term physical or mental ill-health / disability or problems related to old age?* The response options available were: ‘No’; ‘Yes, 1–19 hours a week’; ‘Yes, 20–49 hours a week’; and ‘Yes, 50 or more hours a week’, with respondents told: ‘Do not count anything you do as part of your paid employment’.

We use the data on weekly hours of caring as proxy measures for three levels of caring intensity: ‘moderate’ (1–19 hours a week); ‘substantial’ (20–49 hours a week); and ‘intensive’ (50+ hours a week). Both censuses also collected data on (among other variables) age, family status and household composition, and on employment, health and disability status. We have previously analysed 2001 Census data on carers by age (and other variables) using commissioned data and the ‘Sample of Anonymised Records’ (Yeandle & Buckner 2007). Both sources are used here. We also commissioned new data on people aged 50–54, 55–59 and 60–64 from the 2011 Census.^4^

Although the censuses in 2001 and 2011 provide the main source of statistical data on carers in England for our period, they do not provide information on who the carer assisted, or on whether the carer had support in the caring role. For this we use the SCH, a sample survey for the Department of Health conducted once, in 2009/10. It screened 25,000 English households and collected information on 2200 carers. For aspects of our analysis we accessed the SCH dataset in which data on carers are available for the age groups 45–54 and 55–64.^5^

## Previous Research

Past research on carers of working age has consistently found a particularly high incidence of unpaid caring among women and men aged 50–64, showing that although most carers of this age combine work and care, some leave their jobs or reduce their working hours to facilitate caring. Studies have found those who leave paid work often say they wish they had stayed in their jobs; that many who combine work and care say they need additional support to make their caring responsibilities manageable; and that carers outside the labour market frequently say they would like to return to paid work when their caring responsibilities diminish or end, but think it will be difficult to do this (Arksey et al. 2005; Yeandle et al. 2007; DH 2008; HCWPC 2008).

The official analysis of the SCH showed that among carers aged 45–54 and 55–64, 65% and 72% respectively cared for someone in a separate household (HSCIC 2011: 22), and that about one fifth of carers of this age care regularly for two or more people (HSCIC 2011: 37). Among these carers, most reported having a fairly regular pattern of caring, but 30% of those aged 45–54, and 25% of those aged 55–64, said their care varied from day to day or from week to week (HSCIC 2011: 43). Almost half these carers said caring affected their personal relationships, social life and leisure (47% of those aged 45–54; 43% of those aged 55–64) (HSCIC 2011: 58) and most (68% and 71% respectively) anticipated an increase in caring responsibilities in the next five years (HSCIC 2011: 70).

Other research shows that while caring for elderly parents is common, it is by no means the only form of care provided by people aged 50–64. The English Longitudinal Study of Ageing (ELSA), for example, showed that among carers in their 50s in 2006,^6^ only about half (53% of women and 45% of men) cared for a parent or parent-in-law (Vlachantoni 2010: 11), and a survey of 1647 carers aged 18–64 in England, Scotland and Wales found in 2006–07 that carers aged 50–64 were the group most likely to care for a sick or disabled partner or adult child with disability (Yeandle & Buckner 2007; Yeandle et al. 2007; Yeandle & Cass 2013).

Pickard (2008) has argued that the predicted rise in the number of intergenerational carers is likely to be outstripped by a larger increase in the number of older people. Focusing specifically on people aged 30–74 who regularly provide substantial or intensive care for their older parents, she estimated that between 2005 and 2041 their number was likely to rise by almost 28% (from 395,000 to 500,000). She also calculated that caring at this intensity would: rise more sharply among men (+48%) than women (+19%) for people aged 45–64; increase by +49% (+51% for men and +47% for women) among people aged 65–74; and rise faster among single (+58%) than among married or cohabiting people (+10%) for those aged 30–74. Based on this, she argued that England’s social care and employment systems would have to accommodate growing numbers of older workers needing to reconcile substantial and regular unpaid care for their older parents with their own paid work in the formal labour market. Drawing on further analysis, she subsequently estimated that between 2007 and 2032, increases of 18% and 50% respectively were likely to be seen in the number of people aged 45–64 and 65–74 caring for older parents for 20+ hours per week (Pickard 2015: 108).

## Policy Developments in England Relevant to Older Workers with Caring Roles

A policy focus on how to support carers of people who are frail, disabled or seriously ill had already emerged in the UK well before 1995. The first carers’ organisation was established there in 1965,^7^ and in response to its campaigning, and an emerging academic and policy literature on carers, national policy on taxation and income support first addressed carers’ financial circumstances in the 1960s. Official survey data on carers were first collected in 1985 (Green 1988), and in 2001 and 2011 the official national census of population included a question about caring, creating the opportunity to assess change over time between these dates for sub-groups of carers.

The (limited) rights in national social care and employment systems which carers began to acquire in the UK and England in the 1990s and 2000s (further described below) were mainly directed at alleviating pressures on carers which might threaten their health or the sustainability of their care (Clements 2010; Fry et al. 2011; Yeandle and Buckner 2007; Yeandle et al. 2013). They also recognised carers’ contribution to the wider health and social care system and began to address the support needs of people combining paid work with unpaid care. Although the reconciliation of caring and employment had been debated by trade unions, carers’ organisations and some employers since the early 1980s, it did not feature in legislation and public policy until the mid-1990s.

**Fig. 1:**
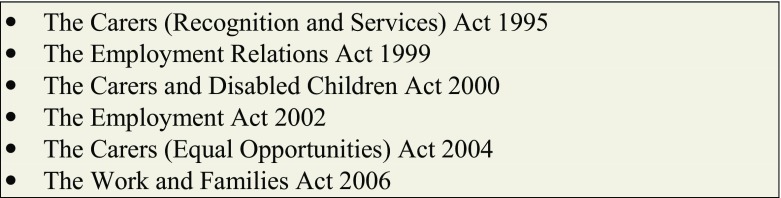
Main legislation relevant to carers and work-care reconciliation: 1995–2006

The main policy developments in England relevant to the growing number of older workers with caring roles were framed by official National Carers’ Strategies in 1999, 2008 and 2010 (HMG 1999, 2008, 2010) and by new legislation (Fig. 1). The *Carers (Equal Opportunities) Act 2004* gave local authorities a statutory responsibility to take a carer’s wishes about paid work into account when conducting a carer’s assessment, and the *Work and Families Act 2006* extended employees’ right to request flexible working to most carers of adults.

In 1999, the (first) National Carers’ Strategy (NCS) acknowledged carers’ need for services and support, noting their difficulties in reconciling work and care. Modest ‘carers’ grant’ funding was subsequently made available to all English local authorities. This stimulated some to develop new services for carers: respite care; emergency carer support; and new programmes of support for carers wishing to remain in work (Clements 2010). Some local authorities used this funding to develop new relationships with local carers’ organisations or to extend or develop the local support services they offered (Fry et al. 2009). The NCS 2008 later strengthened these relationships, and from 2010 a similar approach was adopted by the incoming Coalition Government.

Throughout the period, Carer’s Allowance (CA), described more fully later, also supported growing numbers of people who cared for 35 or more hours each week, primarily those with low participation in paid work or who were outside the labour force.

### Workplace Support for Carers

Carers in England lack any formal rights to paid carer’s leave and have no statutory entitlement to planned time off to make care arrangements when care needs arise.^8^ During the period, however, some employers offered workplace support to their employees who were carers (see below), and advocated the benefits of doing so.

The first, very modest, legal employment rights affecting carers in England were acquired when the *Employment Relations Act 1999* legislated for people with family responsibilities to have the right to take a few days leave for a ‘family emergency’ without fear of dismissal from their job. This entitlement is unpaid, although some employers (at their discretion) offer such support without loss of pay.

The following year, the *Carers and Disabled Children Act 2000* gave carers of a disabled child under age 18 ‘the right to request flexible working’ (provided they had held their job for 26 weeks) and access to a procedure for requesting this, which employers were obliged to offer,^9^ although they could give a ‘business reason’ for rejecting a carer’s request.^10^ From 2007, this limited right was extended to most other carers in the *Work and Families Act 2006*.

Carers outside the labour market who wish to return to paid work can obtain assistance through Jobcentre Plus, the official employment agency, which received additional funding after the 2008 NCS to cover carers’ necessary ‘alternative care’ expenses while attending training to help them return to work. Between December 2009 and February 2011 6620 carers received such ‘work-focused support’; most were women (57%), 40% were over 45, 24% were disabled and 92% were in receipt of state financial support (DWP, 2011). Carers in paid work also acquired limited protection from discrimination at work in the *Equality Act* 2010.^11^

While some employers and trade unions in England had shown interest in supporting work-care reconciliation for carers since at least the mid-1980s (OFW, 1990), real attention to their need for this type of help came first in the voluntary sector. Having highlighted the issue for several decades (Kohner 1993), in the early 2000s the national charity *Carers UK* pressed government to respond to working carers and worked with government and EU officials to establish the relevance of carers to the emerging ‘labour market activation’ agenda (Yeandle & Starr 2007). It then secured EU funding to establish a multi-agency policy and delivery partnership, *Action for Carers and Employment* (ACE), which brought employers, trade unions, policymakers and social care agencies together between 2002 and 2007. ACE examined the barriers carers face in entering, re-entering, or remaining in the labour market and explored the development, design and delivery of alternative care services that enable carers to work (Cook 2007; Yeandle & Starr 2007). It also established the *Employers for Carers* group (EfC) which later became a ‘national membership forum for employers’^12^ and by 2015 had 93 employer members employing over a million people (personal communication).

The organisations which make up EfC stress that they approach work-care reconciliation for employees with caring responsibilities from a business perspective and believe there to be ‘business reasons’ for promoting policies for working carers, citing independent studies and conducting their own surveys and research (EFC & DH 2013). They offer ‘employer-led’ support and services to like-minded employers, and seek to work with government to promote employment policy which supports carers to combine work and care.

EfC’s rationale highlights the *‘demographic drivers’* underpinning its approach: growing numbers of people managing paid work and unpaid caring responsibilities and increased pressures on families and friends to care while also working longer. It argues that most working carers are employees ‘in their prime employment years’ with ‘valuable skills and experience’ and that the one million people who give up (or reduce) their paid work each year (Carers and Ipsos MORI, 2009) represent an annual ‘loss’ for employers as well as for families. It claims a supportive approach to employees who are carers attracts and retains staff, reduces employee stress, cuts recruitment and training costs, increases resilience and productivity, reduces sick leave and absenteeism, improves service delivery, achieves better management of human resources and increases staff morale. Its conclusions reflect independent research findings based on company case studies (Yeandle et al. 2006; Cullen & Gareis 2011; Hamblin & Hoff 2011).

EfC also claims that supporting carers at work yields ‘productivity and performance’ benefits, as employers’ actions can mitigate the negative health, financial and other impacts of intensive caring found in research on carers of working age (Arksey et al. 2005; Buckner & Yeandle 2006; Yeandle and Buckner 2007). It sees supporting carers at work as positive ‘for the wider economy’, noting that better support for working carers can generate substantial additional earnings, tax and national insurance revenue, save on welfare payments (Glasby et al. 2010) and reduce losses to the economy (estimated to be £5.3 billion per year) in the foregone earnings of people who leave work to care (Age UK 2012).^13^ It also makes a ‘*business case for society’,* noting that carers ‘save’ the social care system an estimated £119bn per annum (Buckner & Yeandle 2011).^14^

The government-sponsored ‘Work-Life Balance’ (WLB) surveys conducted in Great Britain between 2000 and 2011 show some forms of workplace flexibility are attractive to carers. Part-time working, working a ‘compressed working week’ and regular home working (the latter between 2003 and 2011) all increased in these years; the Fourth (2012) WLB survey,^15^ which sampled approximately 2000 employees, found that 51% of employees who were carers of an adult (compared with 41% of all employees) said the ‘*availability of flexible working arrangements’* had been *‘very’ or ‘quite’ important* when they had initially decided to accept their current job, and that 67% of carers (compared with 57% of all employees) said flexible working was currently important to them (Tipping et al. 2012). It also found that 56% of employees with caring responsibilities for adults disagreed, or strongly disagreed, with the statement *‘It is not the employer's responsibility to help people balance their work and life*’, a view held most strongly by employees aged 25–59 and those with qualifications. The survey also found that 92% of carers of adults agreed that ‘*having more choice in working arrangements improves workplace morale’.*

In 2011, the Workplace Employment Relations Survey (WERS)^16^ also found, in its survey of employees, that carers and parents made greater use of workplace flexibility than other workers, confirming the WLB survey’s finding that most employees consider flexible working and a supportive attitude towards staff with caring roles beneficial. WERS research with managers in 2011 found three-quarters (76%, compared with 66% in 2004) agreed or strongly agreed ‘*it is up to individual employees to balance work and family responsibilities’,* while only 11% of managers, compared with 17% in 2004, disagreed with this proposition.

### The Evolution of Local Services to Support Carers

The legislation underpinning the development of local carers’ services in England in our period, including the *Carers and Disabled Children Act 2000* and the *Carers (Equal Opportunities) Act 2004,* addressed carers’ needs in ways which proved quite difficult to enforce (Clements 2010). Both Acts contained provisions which strengthened the policy on carers’ assessments initially introduced nationally in the *Carers (Recognition & Services) Act 1995*.

Although the 2004 Act required local authorities to offer carers an assessment of their own needs, and where applicable to take into account their wish to continue in (or re-enter) paid work, its impact was limited as it did not oblige them to provide the services needed to facilitate this (CSCI 2009), and providing services to carers remained discretionary for local authorities throughout our period. Most carers who accessed publicly-funded services from 2004 onwards will nevertheless have done this via a carer’s assessment, whose outcome could be information and advice, a ‘carer-specific’ service (such as respite care or a regular break from caring), or a direct payment. Local authorities could also, if they wished, fund carers’ support groups and help carers to access health checks and training programmes (Yeandle & Wigfield 2011, 2012).

The services provided to the person cared for are also relevant to carers’ ability to combine work and care. One study of over 700 working carers found that while about a third felt their access to services was ‘adequate’ to enable them to work, considerably more said they needed additional services than were satisfied with the support they had (Yeandle et al. 2007). Other research, using data collected in 2009–10, found ‘*a positive association between carers’ employment and receipt of paid services by the cared-for person’*, and that when the cared-for person has home care, the odds of being in employment for male and female carers providing ten or more hours of care per week increase (Pickard et al. 2015).

By the end of our period, official data indicated that 198,000 carers aged 18–64 were being supported by their local authority (in 2011–12); among them, 48% (94,000) received ‘carer-specific’ services and 52% (103,000) ‘information only’. However although the number receiving local authority support increased by 74% between 2008 and 09 and 2011–12, even by the later date, this represented only 5.6%^17^ of all carers of working age (HSCIC 2013).^18^

Data from the SCH *2009–10 (*HSCIC 2011) are consistent with this, and showed that while 66% of carers (of all ages) who supported a relative or person living with them had some contact ‘at least monthly’ with a health and social care professional, only 11% reported receiving a monthly or more frequent visit from a home help or care worker; 5% a visit from a social worker or care manager; 3% a visit from community mental health services; 2% a visit from a health visitor; 2% a ‘Meals on Wheels’ service; and 2% a visit from a voluntary worker (HSCIC 2011:116). Some carers said such help was ‘not needed’ (p118) and 58% said the cared-for person did not attend a day centre, social/support club or other outside activity because they did not want to do so (p 126).

The official data cited here probably underestimate the number of carers with access to some kind of service, for two reasons. First, some local authorities developed local carers’ strategies in the 2000s and worked with voluntary agencies to implement them. Some used their carers’ grant funds to enable these organisations to deliver carer support (including some schemes to help carers manage work and care) without completing a local authority carer’s assessment (Fry et al. 2009), which means carer beneficiaries were not counted in official data. Second, the use of privately purchased services, accessed without reference to a local authority, was already important at the start of our period. The General Household Survey found in 2001 that 650,000 people aged 65 or older had paid for private home help in the previous month (Howse 2007:7), a figure likely to have increased by 2011.^19^

### Financial Help for Carers of Working age

Larger numbers of carers received Carers Allowance (CA), a state financial benefit available to some people caring for 35+ hours per week. Introduced in 1976 as ‘Invalid Care Allowance’, CA was the first financial support for carers. Renamed CA in 2002, it continues to provide financial help to people whose earnings from paid work are reduced by substantial caring responsibilities. The number of CA claimants in England rose consistently during the period studied; by 2012, over half a million carers of working age were receiving it, about three quarters of them women (DWP 2013), and between 2003 and 2013 the number aged 50–64 increased by 61%, reaching 192,990 in 2013 (ONS data from Nomis, July 2014).

While CA moderates the financial impact of caring for carers who give up paid work to care, it plays only a small role in helping them combine work and care. Official administrative data^20^ show that only 10% of CA recipients (12% of women and 5% of men) are in paid work.^21^ To receive CA, a carer must have personal weekly earnings from paid work under £110 (2015 figure, after deductions for tax, care costs while at work and 50% of pension contributions), must be aged 16 or older, and must provide care for 35+ hours per week for a person receiving a state disability benefit. CA is not means-tested against household income, but is taxable, and cannot be claimed by carers studying for 21 or more hours per week. These restrictions mean claimants cannot work full-time (the national minimum wage for adults was £6.19 per hour in 2015^22^) and research shows CA tends to limit carers to low paid, part-time work (McLaughlin 1990; Fry et al. 2011). Consequently most CA recipients are carers who have dropped out of, partially withdrawn from, or never participated in the paid labour force. By 2011–12, CA was costing £1.73 billion per annum and mainly supporting carers outside the labour force who were unable to reconcile work and care.^23^

In sum, then, between 2001 and 2011, carers experienced some improvements in workplace flexibility and gained modest employment protection and rights to support them in combining work and care. A few gained access to local authority services for carers, but it was the support provided to those they care for, especially home care, which increased a carer’s likelihood of remaining in paid work. Financial support for carers played a very limited role in helping carers remain attached to the labour market and for some probably acted as a disincentive to do so. Our policy analysis thus demonstrates a well-established, but dynamic, public policy focus on carers, and that interest in work-care reconciliation for older workers with caring responsibility had begun to receive policy and legislative attention. Nevertheless by 2011 the support, services and rights available to carers combining paid work with their unpaid care remained very modest.

## Results of Statistical Analysis

We now turn to the results of our statistical data on caregiving among older workers. As indicated in the description of our method, we examined changes in their numbers and relationship to the labour market between 2001 and 2011.

### Change in Patterns of Caring among People Aged 50–64, 2001–2011

The number of men and women aged 50–64 living in England rose by just over 1 million between 2001 and 2011, increasing in all three component age bands. The increase was small for those aged 50–54 (+17,000), notable for those aged 55–59 (+210,000) and very large among those aged 60–64 (+776,000), reflecting the ageing of the ‘baby boomer’ cohort born 1947–51.

England in 2001 had 4.9 million carers, of whom 1.7 million (36%) were aged 50–64. By 2011, their total number had risen to 5.4 million, with 1.9 million (also 36%) in the ‘older worker’ age group. The two censuses also showed that demand for care had grown; the number of people aged 85 or older increased from 954,000 to 1.2 million across the decade, and by 2011 there were 9.4 million people (of all ages) self-reporting a ‘limiting long-term illness’, compared with 8.4 million in 2001 (ONS figures).

The increase in caring among the 50–64 age group affected men and women. In 2011, the 1.9 million carers of this age comprised 793,000 men and 1.13 million women, an increase over the decade of 63,000 men and 133,000 women. Detailed analysis using five-year age bands shows, however, that these figures conceal marked variation, and quite different patterns, between age groups. Among people aged 50–54, the number who were carers fell for men (−28,000) and women (−21,000); yet it rose by almost 50,000 in the 55–59 age group (+12,000 for men and +35,000 for women) and increased particularly sharply*,* by +195,000, for those aged 60–64 (+78,000 for men and +117,000 for women).

### Caring Relationships, Gender and age

**Fig. 2:**
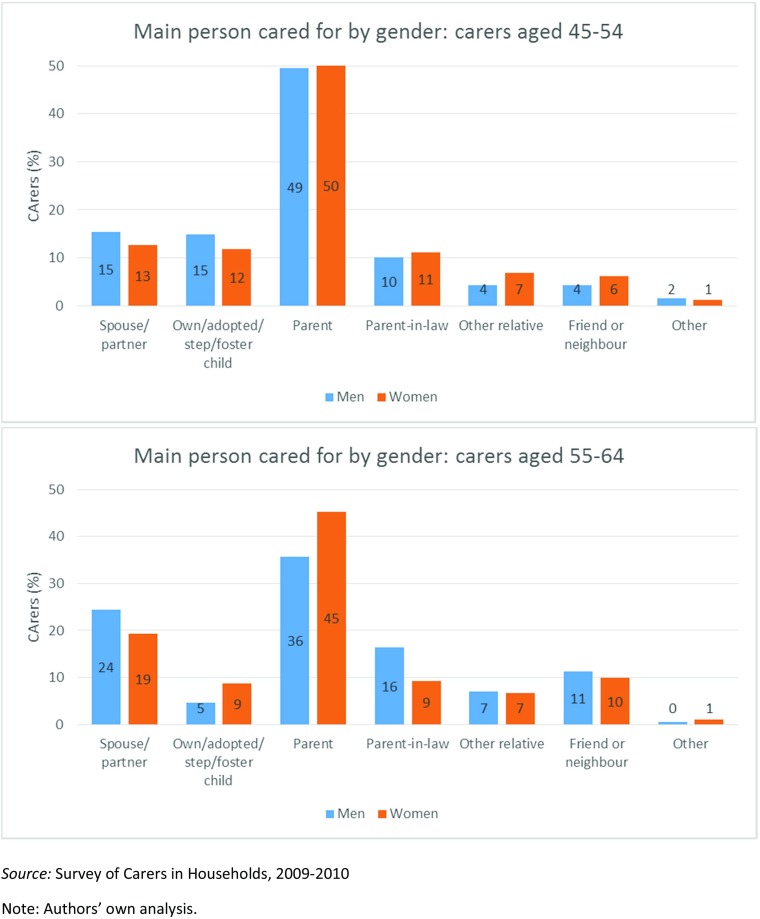
Men and women aged 45–64 with caring responsibility, by relationship to main person cared for, England, 2009–10. *Source:* Survey of Carers in Households, 2009–2010. Note: Authors’ own analysis

Suitable data on caring relationships is available for one data point only, 2009–10, and shows that among 45–54 year old carers, the relationship between male and female carers and those they care for is rather similar. Both men and women cared mainly for a parent (49% of men, 50% of women) or parent-in-law (10% of men, 11% of women) (Fig. 2). Female carers of this age were a little more likely than males to care for a friend or neighbour (6% of women, 4% of men) or for a relative outside their immediate family, i.e. not a parent, child or spouse/partner (7% of women, 4% of men). Males of this age were a little more likely to care for a spouse (15% of men, 13% of women) or for a child (15% of men, 12% of women).

These patterns are somewhat different among 55–64 year old carers, however. At this age, fewer carers of both sexes (36% of male and 45% of female carers) were providing parental care. Care of a parent-in-law was considerably higher (16%) for male carers, although a little lower (9%) for their female counterparts. In this older age group, care of a spouse or partner was higher for carers of both sexes (24% of men, 19% of women); higher for male carers (at 7%) for a relative beyond the immediate family; and lower, for both sexes (down from 15% to 5% for men, and from 12% to 9% for women) for carers of a child (HSCIC 2011).

### Intensity of Caring

**Fig. 3:**
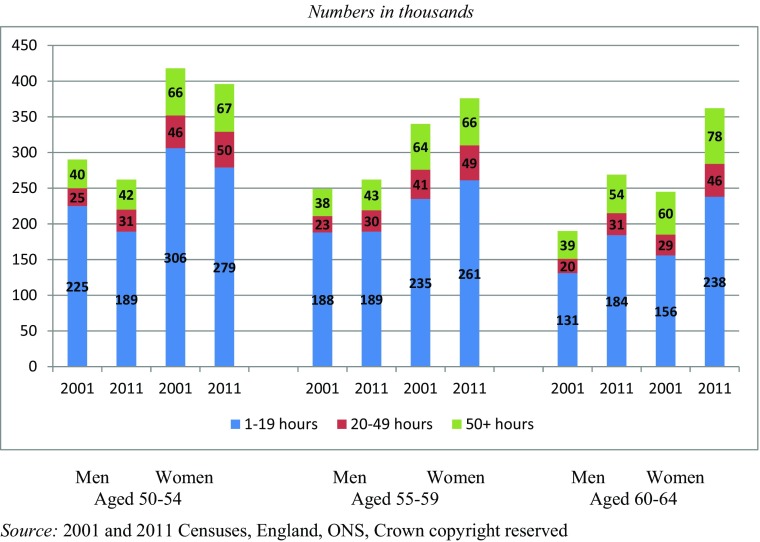
People aged 50–64 who were carers by sex, level of caring intensity, and 5-year age band: 2001 and 2011

The Census data available for 2001 and 2011 show there were notable changes in the intensity of caring between these dates (Fig. 3). For men, the proportion of people providing ‘substantial’ and ‘intensive’ care increased in all three 5-year age bands. By 2011, 4.8% of men aged 50–64 were caring at these levels, compared with 4.4% a decade before, with increases seen in all three age groups: age 50–54 (up from 3.9% to 4.4%); age 55–59 (up from 4.5% to 4.9%) and age 60–64 (up from 5.0% to 5.5%). For women the picture was similar. The share of all women aged 50–64 who were caring at the substantial or intensive level increased from 7.1% to 7.4% between 2001 and 2011, and was seen in all three age groups: 50–54 (up from 6.6% to 6.9%); 55–59 (up from 7.5% to 7.7%); and 60–64 (up from 7.4 to 7.7%) (Office for National Statistics, Census 2001; 2011).

These figures are quite small, but produce a significantly different distribution of carers by caring intensity for men in their fifties. By 2011, 44% of male carers aged 50–54 were caring at the substantial or intensive level, compared with 36% a decade earlier; the figures for those aged 55–59 were 44% compared with 39%. A similar change over time in this indicator was not seen for male carers aged 60–64 or for female carers (in any of the age bands).

Among those caring at the moderate level, however, numbers in the 50–54 age group fell for both sexes: the percentage caring at this level also dropped from 13.5% to 11.3% for men and from 18.0% to 16.3% for women. In the 55–59 age group the number and percentage caring at this level also fell for men (from 13.7% to 12.9%), but for women of this age the drop in numbers represented a small increase in the percentage caring at this level (up from 16.8% to 17.3%). For those aged 60–64, the numbers and percentage of carers rose for both sexes, the latter increasing from 11.2% to 11.9% for men and from 12.9% to 14.8% for women.

From a policy perspective, any large increase in the number of people likely to require support is important, whether the support needed is a publicly funded local service, financial compensation for foregone wages, or a supportive or flexible response from an employer or private service provider. For policymakers, therefore, the marked increase in the period in the number of carers caring at the ‘substantial’ level are of particular interest: for men, +23%, +29% and +55% for those aged 50–54, 55–59 and 60–64 respectively, and for women +9%, +20% and +61% respectively for those in these same age groups.

By far the greatest change was in the number of men and women aged 60–64 who were carers in 2011 (compared to 2001). While this was partly related to an increase in the overall population of this age, which rose by 33% (+777,000 people), the changes were especially sharp for men: +41% at the ‘moderate’ level; +55% at the ‘substantial’ level; and +38% at the ‘intensive’ level. They were also important for women of this age: +53% at the ‘moderate’ level and +61% at the ‘substantial’ level, although lower (+29%), and at a slightly lower level than the population increase, for those caring intensively.

To gain a fuller understanding of the changes affecting 60–64 year olds, we now consider changes in their employment status using self-reported economic activity data from the Census SAR (Sample of Anonymised Records) for 2001, and newly commissioned data from the 2011 Census.^24^ The period saw large changes for men and women, including for those without caring roles.

### Changes in the Employment Status of 60–64 year old Carers, 2001–2011

Among non-carers, 60% of men aged 60–64 had paid work by 2011 (compared with 50% in 2001) and 38% of women (compared with 27%). More male non-carers said they were ‘retired’ from paid work in 2011 (27% compared with 24% in 2001), whereas among female non-carers there was a contrary change, only 55% saying they were ‘retired’ in 2011, compared with 62% in 2001. Among non-carers the percentage of men aged 60–64 who said they were ‘permanently sick or disabled’ also declined sharply (from 19% to 9%) with a similar change among women, albeit at a smaller scale (from 6% to 3%).

Changes in the employment status of 60–64 year olds with caring responsibilities between 2001 and 2011 were particularly notable (Table 1). For those caring at the moderate level the percentages in paid employment were similar to those for non-carers; up sharply from 53% to 61% for men and from 28% to 41% for women. As might be expected, those with substantial or intensive caring roles had lower levels of paid employment, but for them the change over time was also large: for men with substantial caring, up from 42% to 49%, and for those caring intensively from 25% to 33%. For women the increases were from 22% to 34% for substantial and from 14% to 23% for intensive carers. Increases were seen in all types of employment (i.e. in part-time and full-time employment and in self-employment).^25^

**Table 1:** Economic activity status of 60–64 year olds by weekly hours of care, England, 2001 & 2011

Percentages									
People aged 60–64		Men: weekly hours of care				Women: weekly hours of care			
		No care	1–19 hours	20–49 hours	50+ hours	No care	1–19 hours	20–49 hours	50+ hours
All in paid work*	2001	50	53	42	25	27	28	22	14
All in paid work	2011	60	61	49	33	38	41	34	23
Retired	2001	24	32	25	25	61	62	63	68
Retired	2011	27	31	33	39	55	55	60	68
Permanently sick/ disabled	2001	19	11	20	30	6	2	6	7
Permanently sick/ disabled	2011	9	4	9	13	3	1	2	3
Looking after home/ family	2001	0	1	6	13	4	5	6	9
Looking after home and family	2011	0	0	4	9	2	2	3	5
Unemployed and other economically inactive	2001	7	5	7	7	3	3	2	2
Unemployed and other economically inactive	2011	5	4	6	6	2	2	2	2

2001 Census SAR (Sample of Anonymised Records) and 2011 Census Commissioned Tables, Crown copyright

Columns may not add to 100 because of rounding

*This category includes full-time and part-time employees and those self-employed on a full-time and a part-time basis

Among those caring at the moderate level, the proportion who were retired barely changed for men (down from 32% to 31%) but fell sharply among women (from 62% to 55%). The proportion saying they were permanently sick or disabled also declined (though here the changes were smaller than for non-carers).

These figures show that combining paid work with substantial care responsibilities became much more common for 60–64 year olds over the decade. The numbers (for both sexes) almost doubled, from 18,500 to 34,600. The figures for 2001 and 2011 are all derived from the census of population, albeit from different datasets within it. Direct comparison is appropriate, however, as the 2001 figures are based on random samples of the total population (with estimates produced by scaling taking into account the appropriate sampling fraction) and the 2011 data is based on whole population data. The increase in combining work and care for men occurred mainly in full-time employment (up from 24% to 28%), but was seen for women in both full-time and part-time employment (rising from 5% to 11% for the former, and from 14% to 18% for the latter).

At both census dates, most people aged 60–64 who were caring intensively were outside the paid labour force. In 2001, only a quarter of men in this category had paid work (25%; 20% working full-time and 5% part-time) and even fewer women (14%; 4% working full-time and 10% part-time). By 2011 these figures were much higher, with one third of 60–64 year old men who were caring intensively also in paid work, and almost a quarter of women. The increase for men was from 25% to 33%, with increases in full and part-time employment and in self-employment. For women the rise was from 14% to 23%, with increases across all employment types (particularly work for an employer), the percentages doubling from 3% to 6% for full-time employees and rising from 9% to 13% for part-time employees. These changes occurred in the context of a sharp fall in the percentage of men aged 60–64 caring intensively while permanently sick or disabled (down from 30% to 13%) and a similar fall, from a lower base, for women in this situation (down from 7% to 3%).

A consequence of these changes was that by 2011 English employers had many more workers aged 60–64 who were carers than in 2001: 115,586 full-time employees and 94,115 part-time employees, compared with 63,570 and 49,870 in 2001. When all carers in paid work aged 50–59 and self-employed carers aged 60–64 are added, between 2001 and 2011 the total figure for caregiving 50–64 year old workers increased by 121,150 full-time and 90,900 part-time workers, together an additional 212,050 carers in paid work, within the overall growth in the numbers of carers aged 50–64 from 1.7 million in 2001 to 1.9 million in 2011.

Figures for carers aged 50–59 and 60–64 in paid work are shown in Table 2. These show net numerical increases between 2001 and 2011 of +82,000 carers in paid work aged 50–59, and +130,000 aged 60–64. The net figures for men were −1000 (men aged 50–59) and +58,000 (men aged 60–64); for women, +83,000 (women aged 50–59) and +70,000 (women aged 60–64). The net decrease in the number of male carers aged 50–59 in paid work is wholly attributable to a fall in men caring for 1–19 h per week (−18,000), as among those caring for 20–49 and 50+ hours per week the numbers increased by 11,000 and 6000 respectively.

**Table 2:** Carers aged 50–64 in paid work, by gender and weekly hours of care provided: England 2001–2011

(Thousands)								
Age band	Year	All carers	Men: weekly hours of care			Women: weekly hours of care		
			1–19 h	20–49 h	50+ hours	1–19 h	20–49 h	50+ hours
50–59	2001	870	340	31	36	373	45	45
	2011	952	322	42	42	429	60	57
60–64	2001	146	68	8	10	45	7	9
	2011	276	112	15	17	99	15	17

Census 2001 SAM and Census 2011 Commissioned, England, Crown copyright

It is not possible to differentiate the number of carers aged 50–59 in paid work by employment status (employed / self-employed) as these data are not available. The Census SAR (Sample of Anonymised Records) only provides this data for people in the age band 45–59, and although the Census SAM (Small Area Microdata) provides data on people aged 50–59, it does not distinguish those who are employed / self-employed.

## Discussion

In addressing our research questions, we have seen that patterns of caring and labour force participation for people aged 50–64 changed in important ways between 2001 and 2011. Far more men and women were combining a paid job with a substantial or intensive caring role in 2011, and there were big increases in the numbers and percentages of people caring at these levels, especially alongside full-time jobs. These changes have important implications for employers, service providers and policymakers.

We have no comparable data for our two time points on who these carers were supporting, but SCH data show older workers are more likely to have caring responsibilities in their own household (often for a spouse or partner) at later ages. Care elsewhere (often for an older parent or partially independent adult child with disability) also continues to be important, especially for women. A key shift between 2001 and 2011 was towards caring at greater intensity, and it thus seems reasonable to conclude that the challenges working carers face are growing and that further development of the modest support for work-care reconciliation described in our policy analysis, in workplaces, local communities and in employment, social care and welfare systems, is needed.

The increases in combining caring with paid work, especially among 60–64 year olds, are related to changes in health, in the demand for care and in employment (and pension) arrangements.^26^ In 2011, fewer 60–64 year old carers (especially men) reported poor health, or permanent sickness or disability, a picture consistent with broader trends in population ageing and with evidence about improved medical treatments and healthcare.

Changes in demand for care were driven by increased longevity and the rising incidence of living with disability or serious illness at younger ages. Survival rates and life expectancy have improved, increasing the number of people with a family member requiring care, especially at midlife. For our 60–64 year olds, these changes have driven increases in care outside the household (for frail elderly parents, who often live alone) and in ‘co-resident’ care (for sick or disabled spouses, sons and daughters) (Yeandle & Kröger 2013).

Other changes include women’s stronger attachment to the labour market (Spence 2011), men’s and women’s longer working lives (shaped partly by the need to build pensions for a longer retirement), changed attitudes toward older employees, and shifting perceptions of how transitions to retirement can be managed (Yeandle 2005).

As described, the legislative framework for working and caring changed in some important ways between 2001 and 2011. Employment rights for carers in England remain weak, but workplace practices have been changing, and include greater use of part-time work and increased flexibility in how, where and when work is delivered. Meanwhile, greater attention to carers in health and social care policy in 2001–11 increased general awareness of caring as a mainly familial role affecting men as well as women.

Subsequent policy developments also merit comment as an already dynamic policy environment continues to change. The *Children and Families Act 2014,* effective from June 2014, removed the requirement for an employee invoking the right to request flexible working to be a carer (or parent). Some believe that in ceasing to be contingent on caring or parental responsibilities, this right may become easier for carers to invoke.

The *Care Act 2014,* a major change to the law on social care in England, from April 2015 made local authorities responsible for assessing a carer’s needs for support, in all cases where the carer ‘*appears to have such needs’*. It requires them not only to assess those needs but also to ensure they are met (if the carer is eligible for support), a right for which carers had long campaigned (Clements, 2014). Their assessment must take into consideration ‘*whether the carer is able or willing to carry on caring,* (and) *whether they work or want to work*’, and must lead to agreement of a support plan through which the carer’s needs can be met. The impact of this development remains to be seen. It is of interest, however, that the new legislation gives local authorities considerable discretion in what support they offer, and in whether they decide to charge carers (with sufficient means) for the support or services offered through the support plan. Some believe this could help stimulate a more effective market for care services, and create more and higher quality options from which working carers could benefit. Others fear it may lead to further financial pressure on carers if previously free support services become chargeable (Carers UK 2014; DH 2015).

In conclusion we may note that carers in different circumstances are likely to benefit in different ways from the various types of work-care reconciliation support described in this article. To fully assess this, some improvements in data are needed: to maximise analytical potential, these should be better aligned to available census data, and trend data on uptake of entitlements, differentiated by occupation, industry and employment status are required. Increasing numbers of older workers providing care at substantial or intensive levels, as shown in the analysis presented, are likely to mean a need for: more support and flexibility in the workplace (something employers of many different types can offer, as exemplified by members of the Employers for Carers forum); more reliable, tailored services for the person cared for, adaptable as needs change; and a framework of rights and entitlements in employment, welfare and social care systems which supports them to make choices enabling them to provide care without putting their own health, financial wellbeing or social support at risk. Additional options, such as paid carers’ leave in appropriate circumstances (already available in some other jurisdictions), a wider range of quality services in support of people with disabilities and serious illness, and more accessible support in managing practical issues in diverse caring situations, are all also likely to be required.

## Footnotes

1 ‘Carers’ is used rather than ‘informal carers’ as it is the term used in policy and legislation in the UK / England. In law, it includes all people who provide care for others as part of a relationship with the person(s) needing care or support, and excludes people who provide their care as part of paid professional responsibilities or as volunteers for charitable or similar organisations. Statistical data in the article use the (similar but not identical) definition of carers employed in the Census, as described on page 4.

2 From this point, data refer to England (not the UK). Through the *Scotland Act 1998*, the *Northern Ireland Act 1998* and the *Government of Wales Act 1998*, health and social care policy in Scotland, Northern Ireland and Wales became the responsibility of the national administrations in these countries of the UK.

3 In the period, one of the authors (SY) advised a parliamentary inquiry on carers and participated in official task groups on caring and employment (2007, 2012) and relevant carers’ advocacy groups.

4 The commissioned data cover: intensity of caring (weekly hours of caring in three bands); gender; age (in bands 50–54, 55–59; 60–64); and standard ILO measures of economic activity.

5 For technical reasons, data from the Survey of Carers in Households (SCH) are not available in the 5-year age-bands used here to analyse census data.

6 The definition of carers in the ELSA is broader than in the census or in the SCH, as it also includes care for a non-disabled grandchild.

7 This organisation eventually became the charity Carers UK (Yeandle et al. 2013).

8 A few employers offer this as an employee benefit or part of flexible working arrangements.

9 In its original formulation, this involved meeting the employee within 28 days to discuss the application.

10
www.gov.uk/flexible-working/making-a-statutory-application.

11 The complainant in this case was the mother of a disabled child who had been unfairly treated in her place of work, *Coleman v Attridge Law* (2008) C-303/06.

12
https://www.employersforcarers.org/about-us/our-members.

13 In a separate analysis, Pickard (2012) estimated the public expenditure costs of carers leaving employment to be £1.3bn per annum.

14 This figure increased to £132 billion in a revised and updated analysis published in 2015 (Buckner & Yeandle 2015).

15 The Fourth Work-Life Balance Survey was conducted in 2011: data are based on 2767 respondents.

16 The WERS survey has ‘mapped employment relations’ in Britain since 1980, collecting data from managers (2680 responses), employees (21,981 responses) and employee representatives (1002 responses). WERS 2011 findings are compared with data for 2004 in Van Wanrooy et al. 2013.

17 Authors’ calculation, derived from HSCIC 2013.

18 Data for earlier dates are not directly comparable.

19 The GHS did not subsequently collect comparable data.

20 The Department for Work and Pensions collects administrative data on CA; data for 2009 were analysed in a DWP-commissioned study of CA customers (Fry et al. 2011)

21 65% of people who claim CA reside with the person they care for; 64% are women and 36% are aged 50–64.

22 Rate applicable, and legally enforceable, in 2013. A person aged 21+ would thus earn £100 in a little over 16 h.

23 Figure for UK, http://www.guardian.co.uk/news/datablog/2013/jan/08/uk-benefit-welfare-spending.

24 5 year age band data for people aged 50–59 are not available from the 2001 Census SAR.

25 ‘All in paid work’ in Table 1 includes people ‘employed FT’, ‘employed PT’, ‘self-employed FT’ and ‘self-employed PT. Analysis by employment type shows employment rates increased 2001–11 in all four categories, for men and for women, in all four categories (‘no care’; ‘caring 1–19 h pw’; ‘caring 20–49 h pw’; and ‘caring 50+ hours pw’), with just two exceptions. The proportion of men caring for 20–49 h pw who were self-employed FT remained stable (9%) and the proportion of women with no caring responsibilities who were self-employed FT remained stable (2%).

26 It is possible that the rise in State Pension Age (SPA) for women (legislated for in the *Pensions Act 1995* and phased in from mid-2010) affected the number of female carers aged 60–64 in employment in 2011 (causing some women who would otherwise have retired on their 60th birthday to remain in work until they could draw their state pension at 60.5 years). This cannot be definitely stated, however. To assess how many (and which) carers were affected by the SPA changes, single year of age census data on carers and date of birth data on the women in the relevant year would be needed; these are not available.
